# Identification and functional verification of key genes involved in alkaloid biosynthesis in *Pinellia ternata*

**DOI:** 10.3389/fpls.2026.1737389

**Published:** 2026-04-28

**Authors:** Yanyan Pei, Zhijing Wang, Cheng Chen, Nan Xu, Chenjia Shen, Yunting Sun, Shuling Wang

**Affiliations:** 1 School of Pharmacy, Zhejiang Provincial Key Laboratory of Anti-Cancer Chinese Medicines and Natural Medicines, Hangzhou Normal University, Hangzhou, Zhejiang, China; 2 Key Laboratory of Organosilicon Chemistry and Material Technology of Ministry of Education, Hangzhou Normal University, Hangzhou, China; 3 Key Laboratory of Organosilicon Material Technology of Zhejiang Province, Hangzhou Normal University, Hangzhou, China; 4 Laboratory of Chinese Medicine Preparation, Shandong Research Academy of Traditional Chinese Medicine, Jinan, China; 5 College of Life and Environmental Sciences, Hangzhou Normal University, Hangzhou, Zhejiang, China; 6 Hangzhou TCM Hospital Affiliated to Zhejiang Chinese Medical University, Hangzhou, Zhejiang, China

**Keywords:** alkaloid, gene expression, high-throughput technology, *Pinellia ternata*, Pinelliae Rhizoma

## Abstract

**Introduction:**

Pinelliae Rhizoma (PR), the dried tuber of *Pinellia ternata* (Thunb.) Breit, has been used medicinally in China for centuries. PR contains several active ingredients, including alkaloids, which serve as important indicators of PR quality. However, the complete alkaloid biosynthesis pathway in *P. ternata* has not yet been elucidated.

**Methods:**

In this study, LC-MS analysis was used to identify two cultivated varieties of *P. ternata* with distinct alkaloid profiles. Transcriptome sequencing was performed to obtain unigenes and annotate secondary metabolic pathways. Key enzyme and transcription factor genes involved in alkaloid synthesis were further screened, and functional verification was conducted via gene overexpression in *P. ternata* callus.

**Results:**

Transcriptomic profiling yielded 63,106 unigenes, many of which were mapped to diverse secondary metabolic pathways. Based on reference sequences, we predicted the putative alkaloid biosynthetic pathways and identified numerous genes encoding key enzymes and transcription factors. Notably, several genes related to monoterpenoid indole alkaloid and benzylisoquinoline alkaloid pathways, such as *MAO*, *NCS I*, *NCS II*, and *TyrAT*, exhibited significant differential expression between the two varieties. Furthermore, overexpression of *MAO*, *NCS II*, and *TyrAT* in *P. ternata* callus significantly altered precursor accumulation and alkaloid metabolism.

**Discussion:**

Our findings establish a molecular framework for understanding the regulation of alkaloid biosynthesis in *P. ternata*, providing essential insights for the quality control of PR. The differentially expressed genes (e.g., *MAO*, *NCS II*, *TyrAT*) and the results of overexpression experiments provide direct evidence for their roles in alkaloid metabolism. This study not only fills the gap in the current understanding of the complete alkaloid biosynthetic pathway in *P. ternata* but also offers essential theoretical insights and technical support for the quality control of PR in medicinal applications.

## 1 Introduction

Pinelliae Rhizoma (PR), the dried tuber of *Pinellia ternata* (Thunb.) Breit, has been a cornerstone of traditional Chinese medicine for more than 2000 years. It exhibits a wide range of pharmacological activities and is employed in the treatment of various ailments, such as typhoid fever, sub-cardiac firmness, hypochondria, sore throat, dizziness, chest distension, coughing, and intestinal tinnitus (Ji et al., 2014). Modern pharmacological research have revealed that PR contains multiple active ingredients (Guo et al., 2022). Due to its abundant active ingredients, PR is widely incorporated into numerous traditional Chinese medicine prescriptions, such as ‘Banxia Xiexin’ Decoction and ‘Banxia Baizhu Tianma’ Decoction (Jiang et al., 2021; Bai et al., 2022; Wang et al., 2022a). Systematic investigation into the quality control and active constituents of PR has emerged as a key research priority to guide its clinical applications.

Alkaloids are the major bioactive components in the *Pinellia* genus (Chen et al., 2023a). Previous studies have demonstrated the anti-epileptic effects of PR-derived alkaloids through enzyme-linked immunosorbent assay and Western-blotting analysis (Deng et al., 2020). Ephedrine, an alkaloid rich in PR, functions as an adrenergic agent and may exert potentially harmful effects on human cells (Fang et al., 2020). Although the complete biosynthetic pathway of alkaloids in *P. ternata* remains to be fully elucidated, several key genes of this pathway have been identified. For instance, 34 unigenes in various alkaloid biosynthetic pathways were identified in *P. ternata* (Guo et al., 2022). Additionally, PHR1, a phosphates starvation responsive factor, has been implicated in the regulation of alkaloid biosynthesis in *P. ternata* (Wang et al., 2022b). Elucidating the alkaloid biosynthesis pathway is therefore essential for future efforts aimed at improving PR quality.

Environmental factors significantly impact the morphologic and physiologic traits of *P. ternata*, including leaf shape, flower color, number of bulbils, and the accumulation of active ingredients, all of which can affect its therapeutic efficacy (Xue et al., 2008; Yang et al., 2014). Previous studies have demonstrated that light quality markedly affects both primary and secondary metabolism in *P. ternata* tubers. Under red light, *P. ternata* produces a larger number of bulbils and exhibits a higher reproductive coefficient compared to those grown under other monochromatic light conditions (Chen et al., 2013). Wild *P. ternata* seedlings are widely distributed throughout China, especially in Sichuan and Henan provinces (Chen et al., 2023b). Consequently, a systematic analysis of how the geographica origin of *P. ternata* tubers influences their medicinal properties is critical for ensuring the quality control of PR.

Transcriptomic analysis has emerged as a powerful tool for identifying functional genes involved in specific biological processes in plants (Stahl et al., 2012; Tyagi et al., 2022; Zhan et al., 2025). For instance, transcriptome sequencing has provided insights into the molecular mechanisms underlying brassinolide-promoted alkaloid biosynthesis in *P. ternata* tubers (Guo et al., 2022). Additional transcriptomic profiling has elucidated the allelopathic effects of phenolic acid on *P. ternata* (He et al., 2022). Recently, integrated metabolomic and transcriptomic analyses have also been employed to identify candidate genes associated with the flavonoid metabolism pathway and corm pigmentation in *P. ternata* (Guo et al., 2023a; Xu et al., 2023). However, the completed alkaloid biosynthesis pathway in *P. ternata* has not been fully elucidated.

Recently, Xue et al. reported a first chromosome-level genome assembly of wild diploid. *P. ternata* (Xue et al., 2024). However, at the time our study was conducted in 2023, this genome resource had not yet been made publicly available. Given the unavailability of the reference genome at the onset of our study, we employed *de novo* transcriptome assembly to comprehensively characterize the transcripts associated with alkaloid biosynthesis. Specifically, we examined differentially expressed genes between two cultivated varieties of *P. ternata* collected from Chuxiong (CX) and Jingzhou (JZ), which exhibit contrasting alkaloid contents. Based on these analyses, we predicted the complete alkaloid biosynthetic pathway in *P. ternata* and functionally validated several key genes via genetic transformation. Our findings provide a theoretical foundation for improving the qualities of *P. ternata* tubers, thereby enhancing their medicinal value.

## 2 Materials and methods

### 2.1 Reagents

Methanol and Acetonitrile for Liquid Chromatography-Mass Spectrometry (LC-MS) analysis were obtained from Honeywell (Morristown, New Jersey, USA). Formic acid was purchased from Sigma. Analytical standards for Guanosine (LOT: J09GB154303), Inosine (LOT: Y13J12C137495), and Trigonelline (LOT: N22HB202063) were provided by Shanghai Yuanye Biotechnology Co., Ltd. The purity of these standards was more than 98%, calculated through High Performance Liquid Chromatography (HPLC) peak area normalization. The methanol and the glacial acetic acid were chromatographically pure, and the water used was ultrapure.

### 2.2 Preparation of sample solution and HPLC analysis

The fresh *P. ternata* tubers, purchased from JZ in Hubei and CX in Yunnan, were rinsed with distilled water, dried in an oven at 60°C until their weight remained constant, and then ground into a fine powder using a mortar and pestle. Subsequently, 0.500 g of the dried powder (retained on the fourth sieve) was transferred into 15 mL EP tubes. Ultrapure water (10 mL) was added, and the mixture was allowed to stand overnight. The sample was then extracted by ultrasonication for 30 min, followed by centrifugation at 12,000 rpm for 10 min. The supernatant was collected. Ultrapure water (10 mL) was added to the residue, and the mixture was sonicated for another 30 min. After centrifugation at 12,000 rpm for 10 min, the supernatants were combined and concentrated to dryness in a water bath at 99 °C using an evaporating dish. Next, the dried extract was transferred to a 10 mL volumetric flask and the solution was shaken well. The solution was centrifuged at 10,000 rpm for 5 min, and the resulting supernatant was filtered through a 0.45 μm membrane to obtain the filtrate, which served as the test solution (Liu et al., 2009; Zhen and Ge, 2017).

Chromatographic column: WATERS UPLC XBRIDGE C18 (4.6 mm × 250 mm, 5 μm); Flow phase: methanol - 0.07% glacial acetic acid solution (6: 94, V/V); Flow rate: 1.0 mL/min; Detection wavelength: 254 nm; Column temperature: 28 °C; Injection volume: 5 μL (Cheng et al., 2012).

### 2.3 RNA extraction and cDNA library construction

For RNA extraction, samples were collected from JZ in Hubei and CX in Yunnan, respectively. Three biological replicates were set up for the samples from each location. The TRIzol reagent (Invitrogen, CA, USA) was utilized for obtaining total RNA in accordance with the manufacturer’s recommendations. Using the Bioanalyzer 2100 and RNA 1000 Nano Lab Chip Kit (Agilent, CA, USA), with a RIN number >7.0, the overall amount and purity of RNA were examined. Utilizing poly-T oligo-attached magnetic beads (Thermo Fisher Scientific, Shanghai, China), 5 μg of all RNA is separated twice to yield poly(A) RNA. Following purification, the mRNA is broken up into tiny fragments at a high temperature with divalent cations. Reverse transcription was applied to create the ultimate cDNA library after the RNA pieces were split, as per the instructions for the mRNA Seq sample preparation kit (Illumina, San Diego, USA). The paired-end libraries had a median insert length of 300 bp (± 50 bp). Following the vendor’s advised procedure, we carried out paired-end sequencing on an Illumina Novaseq™ 6000 at LC Sciences in the United States. The data presented in this study are openly available in the NCBI Short Read Archive with accession number PRJNA989026 and the MetaboLights database with accession number MTBLS8604.

### 2.4 *De novo* assembly, unigene annotation, and expression analysis

Firstly, reads with adapter contamination, low-quality bases, and undetected bases were removed using Cutadapt (Martin, 2011) and custom Perl scripts. FastQC (https://www.bioinformatics.babraham.ac.uk/projects/fastqc/.babraham.ac.uk/projects/fastqc/), which evaluated the Q20, Q30, and GC-content of the clean data, was utilized to confirm the data quality. High-quality, clean data served as the foundation for all downstream studies. The transcriptome was assembled from scratch using Trinity 2.4.0 (Grabherr et al., 2011). Considering shared sequence content, Trinity classifies transcripts into clusters. ‘Gene’ is a somewhat nebulous term that refers to a transcript cluster. The ‘gene’ sequence (Unigene) was chosen as the cluster’s longest transcript.

All assembled Unigenes were aligned with the Nr protein database (http://www.ncbi.nlm.nih.gov/), GO (http://www.geneontology.org), SwissProt (http://www.expasy.ch/sprot/), KEGG (http://www.genome.jp/kegg/), and eggNOG (http://eggnogdb.embl.de/) databases using DIAMOND (Buchfink et al., 2014) with a threshold E-value < 0.00001. TPM calculations were performed on salmon to ascertain the unigene activity levels. With a log2 (fold change) > 1 or log2 (fold change) < -1 and statistical significance (*P* value < 0.05), the differentially expressed unigenes were chosen using the R package edgeR.

### 2.5 Establishment of the callus induction system of *P. ternata*

All plant hormone solutions, including 6-Benzylaminopurine (6-BA), 2,4-Dichlorophenoxyacetic acid (2,4-D) and Kinetin (KT), were purchased from Beijing Coolaber Technology Co., Ltd. The tubers, petioles, and leaves of *P. ternata* were soaked in 75% ethanol solution for 30 s, rinsed three times with sterile distilled water, immersed in a 0.1% mercuric chloride disinfectant solution for further treatment. The time gradient of 0.1% mercuric chloride solution treatment was set as 8, 10, 12, 15, 20 min, and then the plant samples were washed with ddH_2_O and inoculated into callus induction medium (MS + 4.65 µM KT + 9.05 µM 2,4-D) (Wang et al., 2012). For each group, 30 culture bottles were prepared, with three independent explants per bottle. The induction rate, contamination rate and death rate of explants were calculated to determine the best sterilization time and the best explant type. Based on previous studies (Wang et al., 2012), a high-efficiency induction medium for *P. ternata* callus was screened by adjusting the types and concentrations of exogenous plant hormones, using callus induction medium (MS + 4.65 µM KT + 9.05 µM 2,4-D) as the basis. Three different media were used, including A medium (MS + 6.97 µM KT + 4.52 µM 2,4-D), B medium (MS + 6.97 µM KT + 9.05 µM 2,4-D), and C medium (MS + 6.66 µM 6-BA +9.05 µM 2,4-D), to determine the optimal combination of exogenous plant hormones for inducing callus.

### 2.6 Genetic transformation experiment

The genes related to the synthesis of *P. ternata* alkaloids, including *MAO*, *NCS I*, *NCS II*, and *TyrAT*, were successfully linked to the GFP vector (pCAMBIA1300-35S-sGFP-rbcs), resulting in the construction of four recombinant expression vectors: MAO-GFP, NCS I-GFP, NCS II-GFP, and TyrAT-GFP. Fresh and well-growing callus tissues of *P. ternata* were selected and cut into appropriate sizes. The prepared callus tissue was immersed in an infection solution containing *Agrobacterium tumefaciens* EHA105 and kept on a constant temperature shaker (120 rpm) at 28°C for 15 min. The resulting callus tissues were then transferred to a sterilized solid co-culture medium and incubated at 25°C in the dark for 2 d. After co-cultivation, the callus tissues were transferred to a sterilized liquid MS medium containing 2 mg/L 2,4-D, 1.5 mg/L 6-BA, 200 mg/L Timentin (Coolaber, Beijing, China) and 400 mg/L Cefsulodin (Coolaber, Beijing, China) and shaken for 30 min. Then, the callus was washed with sterile water five to seven times, subsequently transferred into a solid medium supplemented with 200 mg/L Timentin and 400 mg/L Cefsulodin. Finally, the callus was transferred into a screening MS medium containing 2 mg/L 2,4-D, 1.5 mg/L 6-BA, and 50 mg/L Hygromycin (Hyg) (Coolaber, Beijing, China). Newly generated callus tissues were harvested for PCR checking. Callus tissues infected with Agrobacterium containing empty vector were used as a negative control.

### 2.7 Metabolite extraction for untargeted metabolomic analysis

Using liquid nitrogen, the collected PR specimens (500 mg each) from diverse sources were crushed into a fine powder after being defrosted on ice. The powder was transferred to microcentrifuge tubes and mixed with 120 μL of pre-cooled 50% methanol. Following being vortexed for 1 minute, the mixture was allowed to stand at room temperature for 10 minutes. 20 μL of the sample were extracted after incubation, and the balance of the extraction mixture was kept at -20 °C overnight. The supernatants were then poured into new 96-well plates after 20 minutes of centrifugation at 4,000 g. After that, the samples were stored at -80 °C until LC-MS. Quality control (QC) samples were prepared by pooling 10 μL of each sample extract.

### 2.8 LC-MS/MS analysis for untargeted metabolomics

According to machine instructions, the LC-MS system was applied to analyze all samples. First, chromatographic separations were performed using an UltiMate 3000 HPLC system (Thermo Scientific). For the reversed-phase separation, an ACQUITY UPLC BEH C18 column (100 mm×2.1 mm, 1.8 µm, Waters, UK) was employed. The column oven was maintained at 35 °C. The mobile phase of the experiment contained solvents A (water with a formic acid concentration of 0.1%) and B (acetonitrile containing 0.1% formic acid), and the flow rate was 0.4 mL/min. The following gradient elution parameters were chosen: 0~0.5 min, 5% B; 0.5~7 min, 5% to 100% B; 7~8 min, 100% B; 8~8.1 min, 100% to 5% B; 8.1~10 min, 5% B. The injection volume was 4 µL for each sample.

Metabolites eluted from the column were detected using a high-resolution tandem mass spectrometer, the TripleTOF6600plus (SCIEX, Framingham, MA, USA). Both positive as well as negative ion modes of the Q-TOF were in active use. The interface heater temperature was set to 650 °C, the pressure of the curtain gas was set to 30 PSI, and Ion Source Gases 1 and 2 were set to 60 PSI. The Ionspray voltage floating was established at 5000 V for the positive ion mode, respectively. The Ionspray voltage floating was set to -4500 V for the negative ion mode. In IDA mode, mass spectrometry data was gathered. The 60 to 1200 Da time-of-flight (TOF) mass range was utilized. The survey scans were taken in less than 150 ms, and if the product ion scans met the criteria of 100 counts per second (counts/s) and a 1+ charge state, up to 12 of them could be gathered. A 0.56 s overall time for each cycle was chosen. Four time bins were added together for each scan at a pulser frequency of 11 kHz. The detector was a four-anode/channel 40 GHz multichannel Time-to-Digital Converter detector.(TDC). For each of the 20 samples collected during the collection process, the mass accuracy was validated. Moreover, a QC sample (a pool of all samples) was obtained after every 10 samples in order to evaluate the reliability of the LC-MS throughout the whole acquisition procedure.

### 2.9 Metabolomics raw data processing and quality control

XCMS software was used for peak picking, peak grouping, retention time correction, second peak grouping, and annotation of isotopes and adducts on the gathered MS dataset. The LC-MS raw data files were converted to mzXML and then analyzed with the R software’s XCMS, CAMERA, and meta X toolboxes. By integrating retention time and *m/z* information, all ions were detected. A three-dimensional matrix was created using the peak intensities as input. This matrix systematically integrated the sample names (observations), ion intensity information (variables), and peak indices (retention time-m/z pairs) to ensure data traceability and consistency.

### 2.10 Metabolite annotation

Metabolites were classified by comparing the accurate molecular *m/z* ratios of the samples with those in the KEGG and HMDB databases. The metabolite could be labeled if there was a mass deviation of less than 10 ppm within the measured and database readings. The molecular formula of each metabolite was then confirmed by isotopic distribution analysis. To corroborate our detection of the metabolites, an internal library of fragment spectra was employed.

Peak intensity data were further preprocessed using Meta X. Features that were found in 80% or less of the biological samples or in fewer than 50% of the QC samples were eliminated. To improve the quality of the data, the remaining peaks with missing values were imputed using the k-nearest neighbor approach. PCA was performed on the preprocessed dataset to evaluate batch effects and detect outliers. To reduce signal intensity drift over time, a reliable LOESS signal correction approach based on QC was applied to the data, taking into consideration the order of injection. Additionally, metabolic features with a relative standard deviation (RSD) > 30% in QC samples were removed. To find variations in the quantity of metabolic buildup between the two sample groups, studen’s t t-tests were used. The Benjamini-Hochberg procedure, a false discovery rate (FDR) adjustment technique, was implemented to adjust the *P-*value for multiple tests. To distinguish between various factors across groups, supervised PLS-DA was carried out using meta X. The VIP score has been determined. 1.0 was utilized as the VIP cutoff value to choose important features.

### 2.11 Statistical analysis

We computed the *P* values for the transcriptome analysis using the FDR. Additionally, we utilized the Benjamini and Hochberg approaches to correct the *P-*values. Version 19.0 of the SPSS program was used for the statistical analysis (SPSS Inc., Chicago, IL, USA). To assess the distinctions between the two teams, an ANOVA was performed.

## 3 Results

### 3.1 Differences in three classic alkaloids between the *P. ternata* tubers from JZ and CX

**Figure 1:**
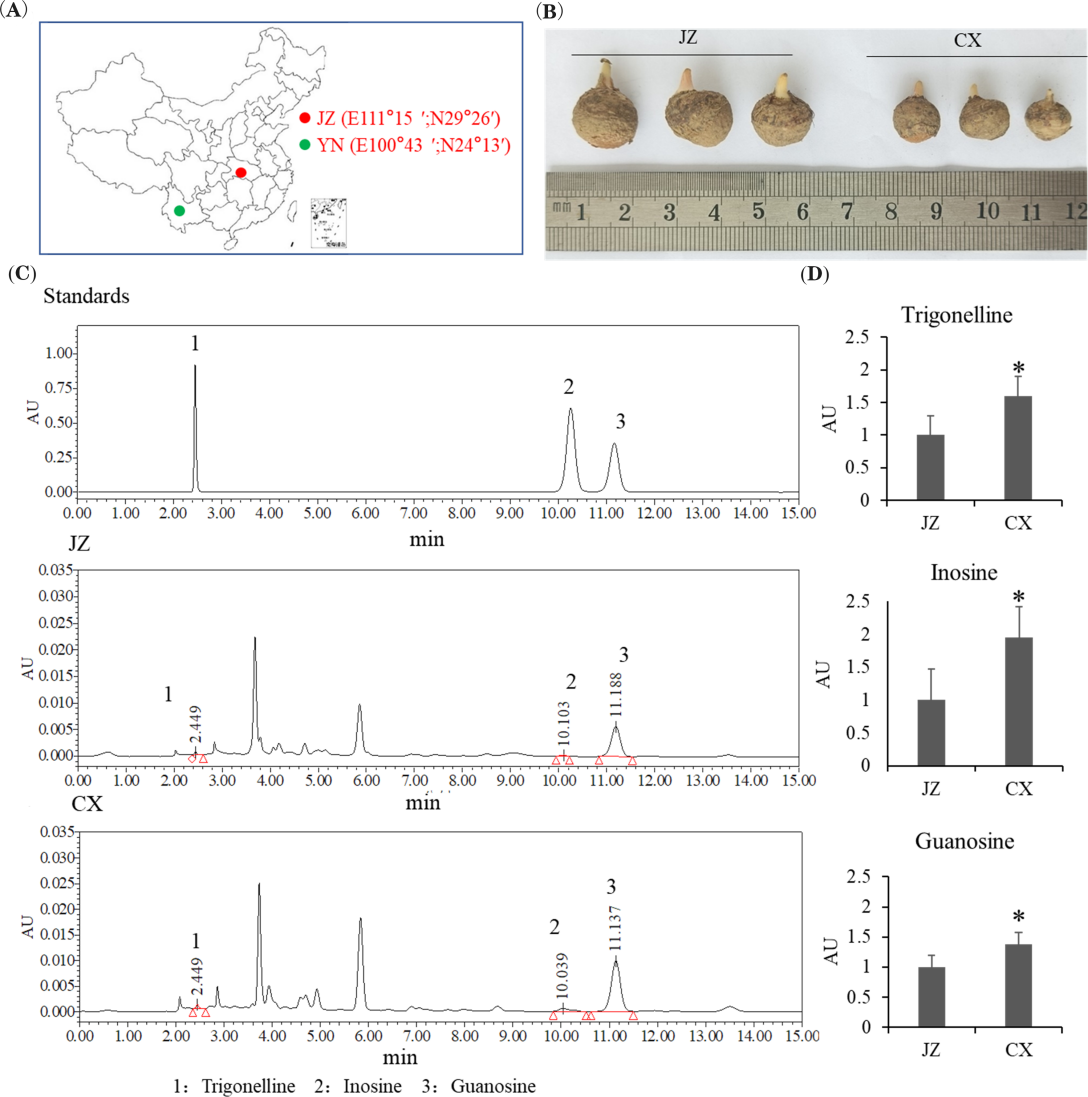
Effect of geographical environments on Pinelliae Rhizoma (PR) from different origins. **(A)** Geographical locations of the two sampling sites in China: JZ (Jingzhou, Hubei Province, E111°15′, N29°26′, red dot) and CX (Chuxiong, Yunnan Province, E100°43′, N24°13′, green dot). **(B)** Morphological comparison of P. ternata tubers collected from JZ and CX. **(C)** HPLC chromatograms of the three alkaloid standards and typical alkaloids in PR samples collected from JZ and CX. 1: Trigonelline; 2: Inosine; 3: Guanosine. **(D)** Quantitative comparison of the contents of trigonelline, inosine, and guanosine in PR from JZ and CX. A P value <0.01 was considered to be statistically significant and indicated by “*”

To analyze the effect of geographical environment on the quality of PR, freshly dried tubers were collected from two cultivated varieties of *P. ternata* in CX (Yunnan, China; E100°43′; N24°13′) and JZ (Hubei, China; E111°15′; N29°26′), respectively (**Figure 1A**). Although these varieties exhibited differences in morphology and size (**Figure 1B**), ITS sequence alignment confirmed their identity as *P. ternata* with >98% homology (**Supplementary Figure 1**), verifying their taxonomic consistency. Three representative alkaloids, including Trigonelline, Inosine, and Guanosine, were selected as quality indicators to evaluate the influence of origin on alkaloid biosynthesis in *P. ternata* tubers (**Figures 1C–E**). HPLC analysis revealed that the levels of Trigonelline, Inosine and Guanosine were significantly higher in the CX tubers than in those from JZ (**Figure 1F**). These results demonstrate that geographical environments markedly influence the morphological traits and alkaloid accumulation profiles of *P. ternata* tubers.

### 3.2 Transcriptome analysis

To investigate the complete alkaloid biosynthesis pathway, a *de novo* assembled transcriptomic analysis was performed. After removing the adapters, the raw reads were quality-checked, generating 34.28 Gb of clean sequence data, consisting of 16.74 Gb from the JZ tubers and 17.54 Gb from the CX tubers. More than 96.32% and 90.85% of clean reads had quality scores at the levels of Q20 and Q30, respectively (**Supplementary Table 1**).

All reads were assembled using Trinity software, resulting in 159,135 transcripts (N50: 965) with a median length of 404 bp, and 63,106 unigenes (N50: 1,020) with a median length of 397 bp (**Supplementary Figure 2A**). Functional annotation revealed that 22,346 unigenes (35.41%) were assigned to the Gene Ontology (GO) database, while 18,305 (29.01%), 17,611 (27.91%), 26,048 (41.28%), and 20,543 (32.55%) were annotated in the Swiss-Prot, KEGG, eggNOG, and Pfam databases, respectively (**Supplementary Figure 2B**). There were 51,309 unigenes (81.31%) with lengths <1000 bp, 8,367 unigenes (13.26%) ranging from 1,000-2,000 bp, and 3,430 (5.44%) with lengths >2,000 bp (**Supplementary Figure 2C**). Species distribution analysis revealed that most unigenes exhibit high sequence similarity to *Elaeis guineensis* (15.09%), *Phoenix dactylifera* (13.03%), and *Nelumbo nucifera* (5.76%) (**Supplementary Figure 2D**).

### 3.3 Classifications of enriched GO and KEGG terms

A total of 22346 unigenes were successfully assigned to at least one GO term. Within the biological process category, the most highly enriched terms were ‘biological_process’ and ‘regulation of transcription, DNA-templated’. ‘Nucleus’ and ‘cytoplasm’ were the two most enriched terms in the classification of cellular components. The most enriched terms within the molecular function category were ‘protein binding’, ‘molecular_function’ and ‘ATP binding’. For the cellular component category, ‘nucleus’ and ‘cytoplasm’ were the two most predominant terms. In the molecular function category, the most enriched terms included ‘protein binding’, ‘molecular_function’,and ‘ATP binding’.

A total of 17,611 unigenes were mapped to multiple KEGG pathways, encompassing six main categories: organismal systems, environmental information processing, metabolism, genetic information processing, human diseases, and cellular processes. Notably, within the ‘Metabolism’ category, the most highly represented pathways included ‘energy metabolism’ (539 unigenes), ‘amino acid metabolism’ (788 unigenes), ‘lipid metabolism’ (687 unigenes), and ‘carbohydrate metabolism’ (1,390 unigenes). Under ‘Genetic Information Processing’, the ‘translation’ term had the most unigenes (1,575 unigenes). For ‘Environmental Information Processing’, the category ‘signal transduction’ was the most highly represented category, with 741 unigenes. For ‘Cellular Processes’ and ‘Organismal Systems’, the terms ‘transport and catabolism’ (699 unigenes) and ‘environmental adaptation’ (495 unigenes) were the most highly represented, respectively.

### 3.4 Identification and classification of the DEGs between the JZ and CX tubers

**Figure 2:**
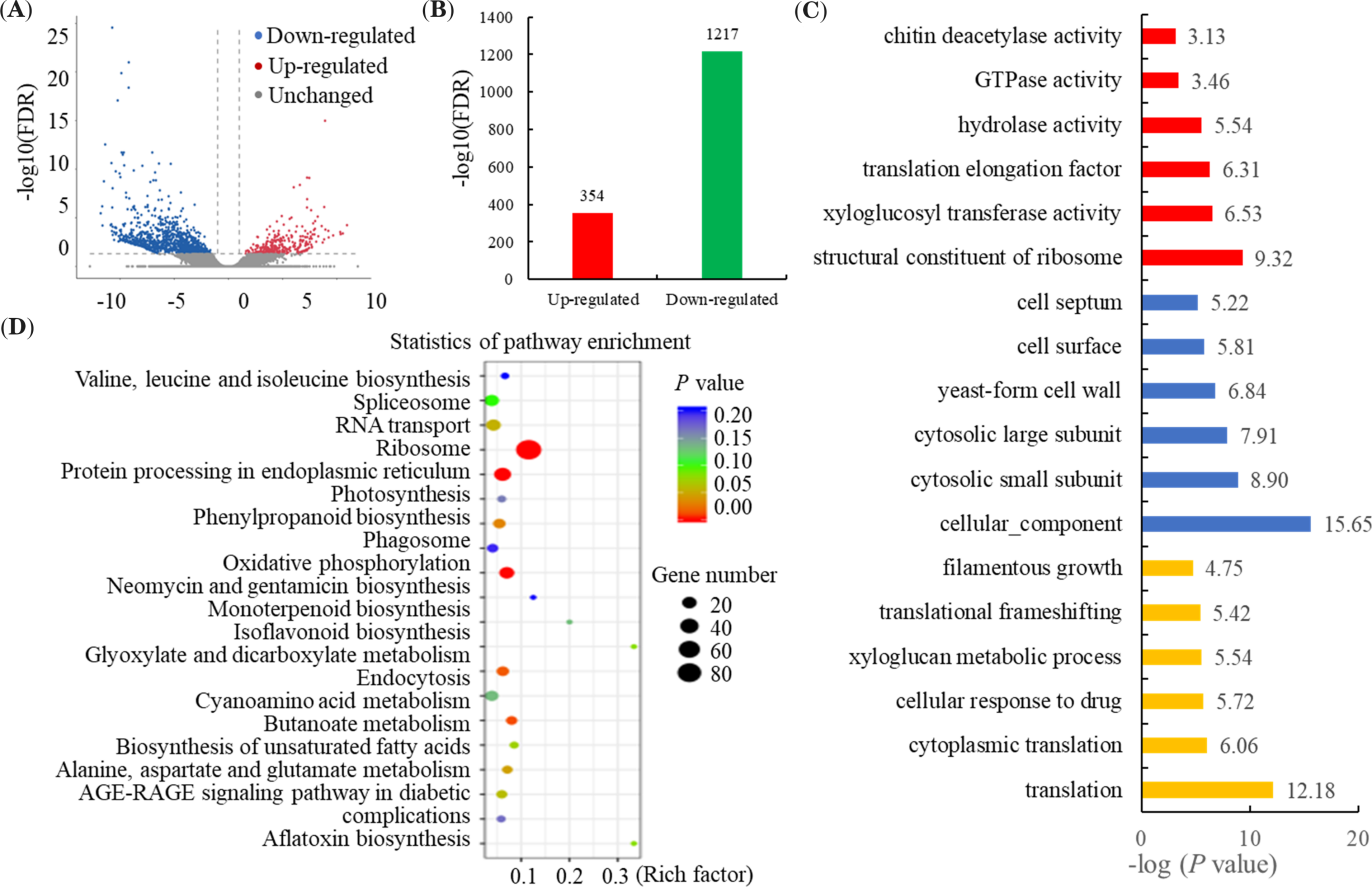
Analysis of the differentially expressed genes (DEGs) between the PR from JZ and CX. **(A)** Significance analysis of the DEGs between the PR from JZ and CX by Volcanoplot analysis. **(B)** The number of up- and down-regulation genes in the JZ/CX comparison. **(C)** GO enrichment analysis of the DEGs between the PR from JZ and CX. **(D)** KEGG enrichment analysis of the DEGs between the PR from JZ and CX.

To identify DEGs between the JZ and CX tubers, the read density was calculated for each gene. A significance analysis was performed on the DEGs and was visualized using a volcano plot (**Figure 2A**). A total of 1,571 DEGs, including 354 up- and 1,217 down-regulated genes, were identified between the JZ and CX tubers (**Figure 2B**).

A total of 609 DEGs were assigned to at least one GO term (**Supplementary Table 2**). ‘Translation’ (*P* = 6.59E-13), ‘cellular component’ (*P* = 2.22E-16), and ‘structural constituent of ribosome’ (*P* = 4.84E-10) were the most enriched GO terms (**Figure 2C**). Then, a total of 441 DEGs were assigned into various KEGG metabolic pathways (**Supplementary Table 3**). The KEGG enrichment analysis showed that the most enriched were ‘ribosome’ (map03010), ‘protein processing in endoplasmic reticulum’ (map04141), ‘oxidative phosphorylation’ (map00190), ‘glyoxylate and dicarboxylate metabolism’ (map00630), and ‘cyanoamino acid metabolism’ (map00460) (**Figure 2D**).

### 3.5 DEGs associated with the monoterpenoid indole alkaloids pathway

**Figure 3:**
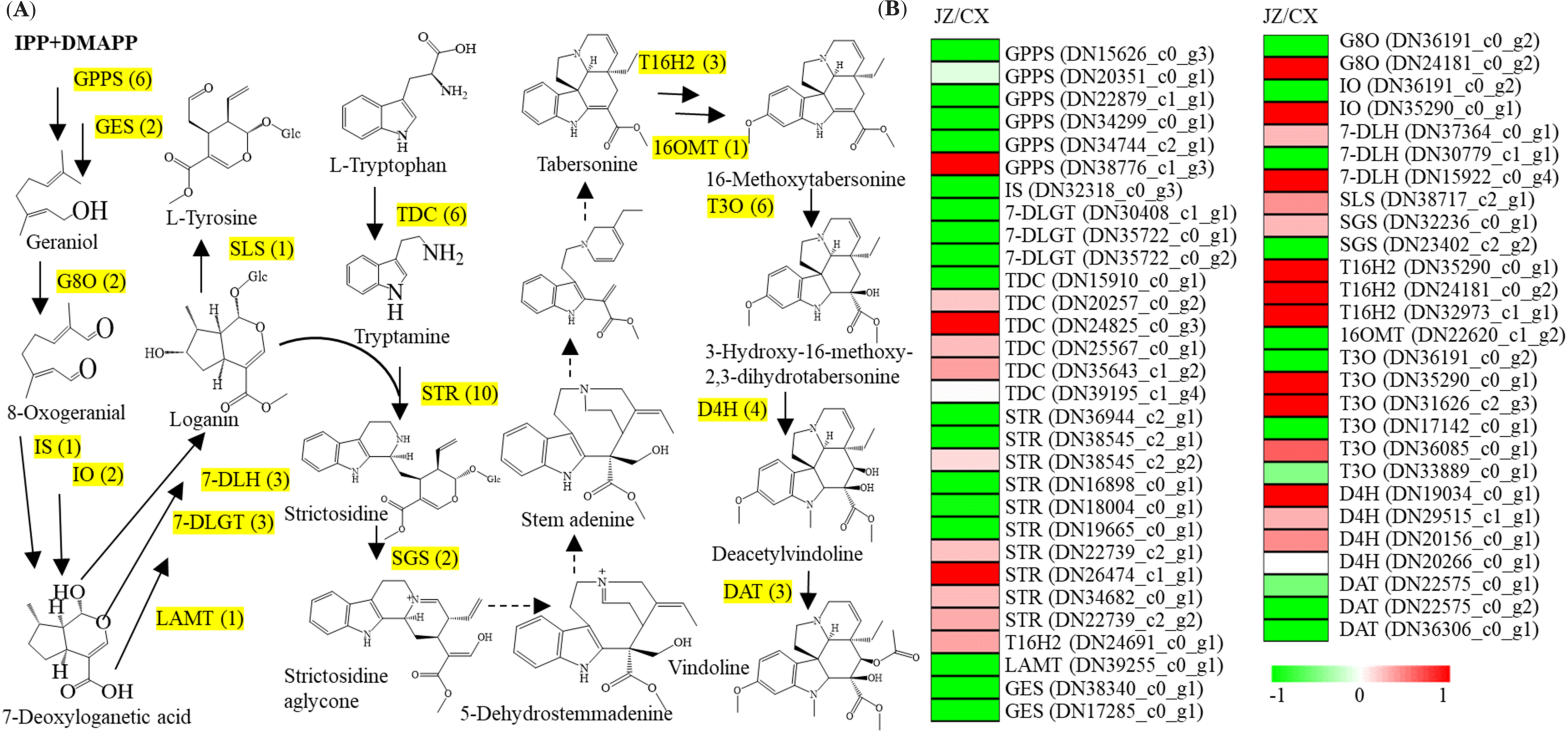
Differential expression of the genes related to monoterpenoid indole alkaloid (MIA) pathway. **(A)** Overview of the classic MIA pathway in *P. ternata.* The numbers in brackets indicated the number of coding genes referring to each enzyme. Enzyme abbreviations are as follows: diphosphate synthase (GPPS), geraniol synthase (GES), geraniol 8-oxidase (G8O), iridoid oxidase (IO), 7-deoxyloganetic acid glucosyltransferase (7-DLGT), deacetylvindoline-4-O-acetyltransferase (DAT), tabersonine 16-hydroxylase 2 (T16H2), Iridoid Synthase (IS), loganic acid O-methyltransferase (LAMT), secologanin synthase (SLS), sterolΔ7-reductase (7-DR), C24-methyltransferase 1 (SMT1), 16-hydroxytabersonine O-methyltransferase (16OMT), Tryptophan decarboxylase (TDC), 7-deoxyloganic acid hydroxylase (7-DLH), strictosidine synthase (STR), strictosidine glucosidase (SGS), tabersonine 3-oxygenase (T3O), and desacetoxyvindoline-4-hydroxylase (D4H). **(B)** Expression changes of genes associated with the MIA pathway. The differential expression levels are showed by heatmaps. The heatmap scale ranges from −1 to +1 on a log_2_ scale normalized expression levels.

Our transcriptomic data identified a number of unigenes associated with the MIA pathway in *P. ternata* (**Figure 3A**). In the MIA pathway, six unigenes that encode geranyl diphosphate synthase (GPPS) were identified. Among the GPPS encoding genes, GPPS1 (DN15626_c0_g3), GPPS2 (DN20351_c0_g1), GPPS3 (DN22879_c1_g1), GPPS4 (DN34299_c0_g1) and GPPS5 (DN34744_c2_g1) were significantly expressed in the CX tubers. Two geraniol synthase (GES) encoding unigenes (DN38340_c0_g1 and DN17285_c0_g1), were prominently expressed in the CX tubers. Two unigenes encoding geraniol 8-oxidase (G8O, DN24181_c0_g2) and iridoid oxidase (IO, DN35290_c0_g1) were annotated, which were predominantly expressed in the JZ tubers. Three 7-deoxyloganetic acid glucosyltransferase (7-DLGT) encoding unigenes (DN30408_c1_g1, DN35722_c0_g1, DN35722_c0_g2) and three deacetylvindoline-4-O-acetyltransferase (DAT) encoding unigenes (DN22575_c0_g1, DN22575_c0_g2, and DN36306_c0_g1) were greatly expressed in the CX tubers. By contrast, three tabersonine 16-hydroxylase 2 (T16H2) encoding unigenes (DN35290_c0_g1, DN24181_c0_g2, and DN32973_c1_g1) were significantly expressed in the JZ tubers. Only one encoding unigene for iridoid synthase (IS), loganic acid O-methyltransferase (LAMT), secologanin synthase (SLS), and 16-hydroxytabersonine O-methyltransferase (16OMT) each were identified. 16OMT (DN22620_c1_g2), LAMT (DN39255_c0_g1), and IS (DN32318_c0_g3) were predominantly expressed in the CX tubers. Tryptophan decarboxylase (TDC) was encoded by six unigenes. Among the six TDC encoding unigenes, four unigenes (DN20257_c0_g2, DN24825_c0_g3, DN25567_c0_g1, and DN35643_c1_g2) were expressed in the JZ tubers. Two 7-deoxyloganic acid hydroxylase (7-DLH) encoding unigenes (DN37364_c0_g1 and DN15922_c0_g4) were predominantly expressed in the JZ tubers, while, one 7-DLH encoding unigene (DN30779_c1_g1) was highly expressed in the CX tubers. Eventually, three desacetoxyvindoline-4-hydroxylase (D4H) encoding unigenes (DN19034_c0_g1, DN29515_c1_g1, and DN20156_c0_g1) were predominantly expressed in the JZ tubers (**Figure 3B**).

### 3.6 DEGs associated with the benzylisoquinoline alkaloids pathway

**Figure 4:**
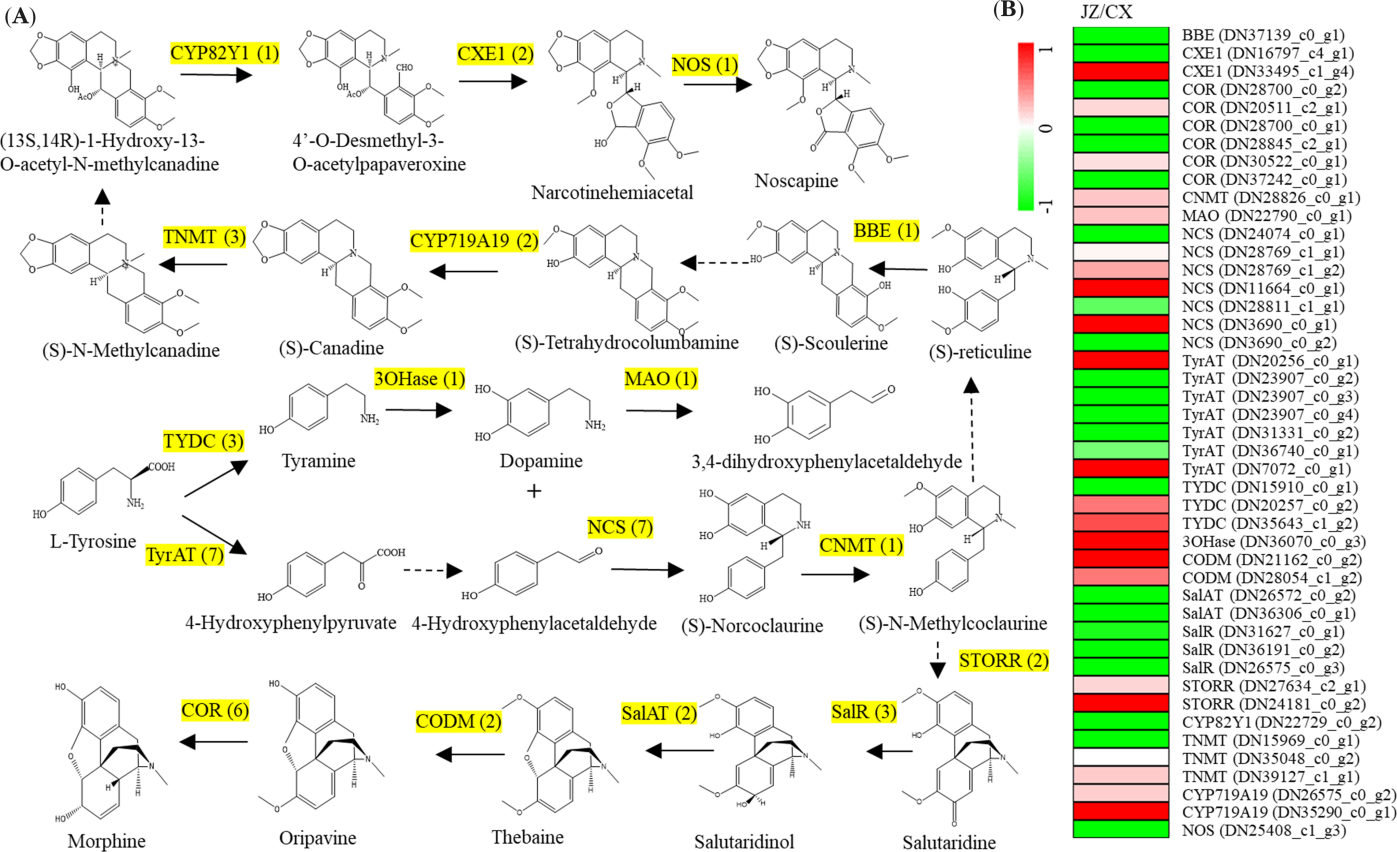
Differential expression of the genes related to benzylisoquinoline alkaloid (BIA) pathway. **(A)** Overview of the classic BIA pathway in *P. ternata.* The numbers in brackets indicated the number of coding genes referring to each enzyme. Enzyme abbreviations are as follows: tyrosine aminotransferase (TyrAT), (S)-norcoclaurine synthase (NCS), Salutaridine reductase (SalR), Codeinone reductase (COR), codeine O-demethylase (CODM), noscapine synthase (NOS), and (S)-tetrahydroprotoberberine N-methyltransferase (TNMT). **(B)** Expression changes of genes associated with the BIA pathway. The differential expression levels are showed by heatmaps. The heatmap scale ranges from −1 to +1 on a log_2_ scale normalized expression levels.

Transcriptomic analysis also identified a number of unigenes associated with the BIA pathway in *P. ternata* (**Figure 4A**). Within this pathway, seven unigenes encoding tyrosine aminotransferase (TyrAT) were identified, two of which (DN20256_c0_g1 and DN7072_c0_g1) were highly expressed in the JZ tubers. Three unigenes encoding tyrosine decarboxylase (TYDC) were annotated, one of which (DN15910_c0_g1) was predominantly expressed in the CX tubers. (S)-norcoclaurine synthase (NCS) was encoded by seven unigenes, of which three (DN24074_c0_g1, DN28811_c1_g1, and DN3690_c0_g1) were primarily expressed in the CX tubers. Two salutaridinol 7-O-acetyltransferase (SalAT) synthase encoding unigenes (DN26572_c0_g2 and DN36306_c0_g1) were predominantly expressed in the CX tubers. Additionally, three salutaridine reductase (SalR) encoding unigenes (DN31627_c0_g1, DN36191_c0_g2, and DN26575_c0_g3) and six codeinone reductase (COR) encoding unigenes (DN28700_c0_g1, DN28700_c0_g2, DN20511_c2_g1, DN28845_c2_g1, DN30522_c0_g1, and DN37242_c0_g1) were greatly expressed in the CX tubers. Interestingly, two codeine O-demethylase (CODM) encoding genes (DN21162_c0_g2 and DN28054_c1_g2) were prominently expressed in the JZ tubers. Berberine bridge enzyme (BBE), noscapine synthase (NOS), and CYP82Y1 encoding unigenes significantly expressed in the CX tubers. In contrast, one tyrosine/tyramine 3-hydroxylase (3OHase) encoding unigene (DN36070_c0_g3) highly expressed in the JZ tubers (**Figure 4B**).

### 3.7 Identification of transcription factors

It has been believed that a number of TFs are involved in the secondary metabolite biosynthesis in plants (Yu et al., 2022). In our study, several TF encoding genes exhibited significant differential expression between the JZ and CX tubers, including three genes for auxin response factors (ARFs), two genes for myelocytomatosis (MYC), one gene for MADS-box (MADS), two genes for basic zipper (bZIP), three genes for basic helix-loop-helix (bHLH), four genes for v-myb avian myeloblastosis viral (MYB), and three genes for ethylene responsive factor (ERF) (**Supplementary Table 4**). All three *ARF* genes significantly upregulated in the JZ tubers. Among the three *bHLH* genes, DN31608_c1_g1 and DN27538_c1_g3 predominantly expressed in the CX tubers, while DN36960_c0_g1 was predominantly expressed in the JZ tubers. Moreover, one *MADS* gene, two *bZIP* genes, and three *ERF* genes were significantly enriched in the CX tubers. For the *MYB* family genes, the expression levels of DN26714_c0_g2 and DN35486_c0_g1 highly expressed in the JZ tubers, while DN24106_c1_g2 and DN19837_c0_g1 were greatly expressed in the CX tubers. For the *MYC* family, DN31484_c2_g1 primarily expressed in the CX tubers, while DN18915_c1_g2 was significantly upregulated in the JZ tubers.

### 3.8 Establishment of the callus induction system of *P. ternata*

**Figure 5:**
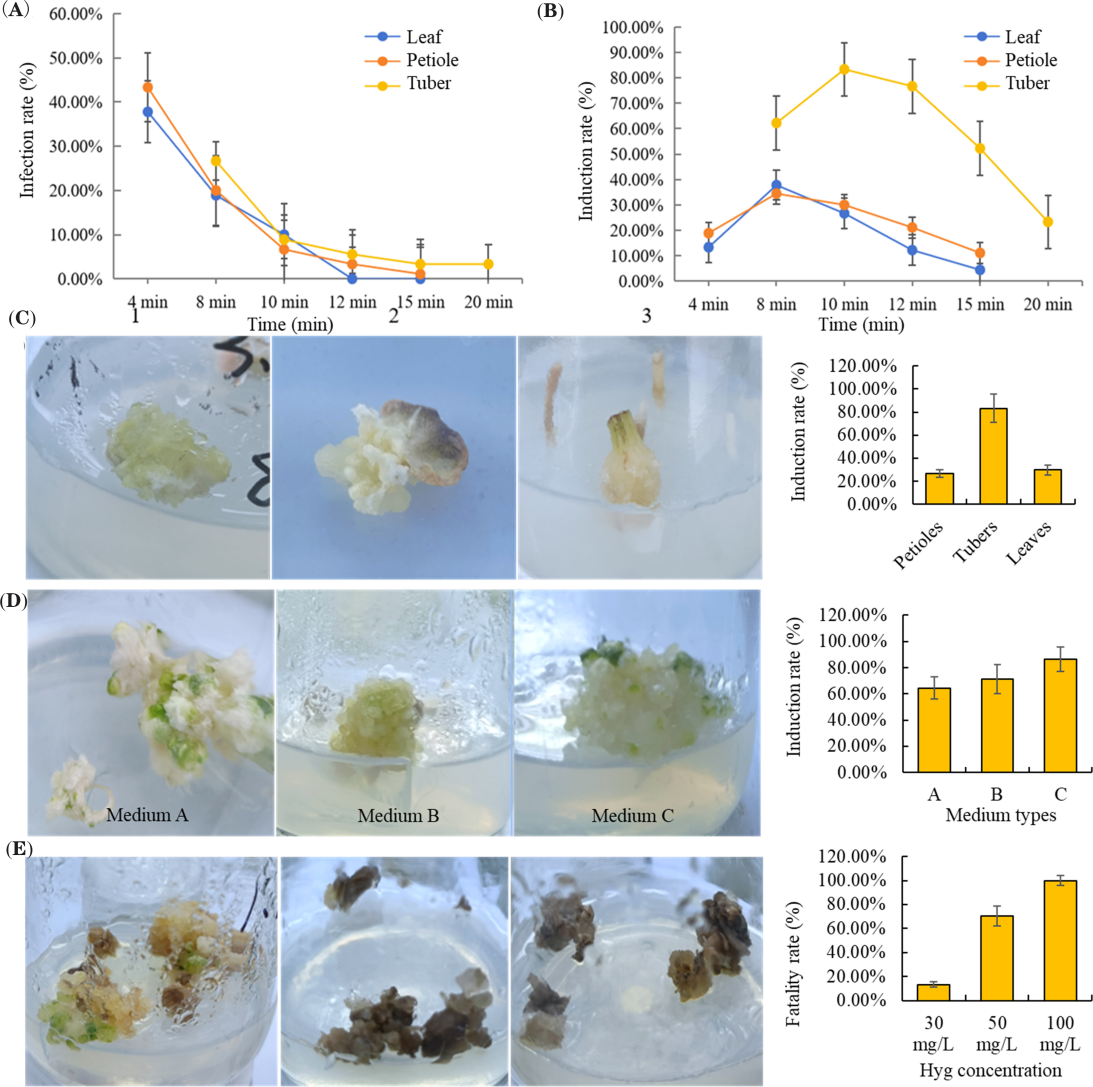
Establishment of the callus induction system of *P. ternata*. **(A)** Analysis of the infection rates of three different explants under different sterilization durations. **(B)** Analysis of the induction rates of three different explants under different sterilization durations. **(C)** The callus induction rates of three different explants under optimal sterilization conditions. **(D)** Selection of the best medium for the callus induction of *P. ternata.***(E)** Determination of the optimal concentration of hygromycin for positive callus selection.

In our study, petioles, leaves, and tubers of *P. ternata* were selected as explants for callus induction. The explants were sterilized using a 0.1% mercuric chloride solution, and the effects of different sterilization durations on the contamination rates of the explants were analyzed. After 4 minutes of sterilization, the contamination rates for leaves and petioles were 37.78% and 43.33%, respectively. After 8 minutes of sterilization, the contamination rate for tubers was 26.67%. The contamination rates for all three sample groups were decreased to below 10% after 10 min sterilization. Thus, treatment with 0.1% mercuric chloride solution for 10 minutes sterilization was determined to be the optimal sterilization condition (**Figure 5A**).

Under optimal sterilization conditions, the callus induction rate using tubers as explants (83.33%) was higher than that of leaves (30.00%) and petioles (26.67%) (**Figure 5B**). Moreover, the induced callus derived from tubers was pale yellow and dense, indicating that the sterilization protocol effectively prevented contamination without adversely affecting callus formation (**Figure 5C**). Based on these results, tubers were selected as the preferred explant for subsequent callus induction experiments.

Although callus was effectively induced by all three tested media, the C-type medium (MS + 1.5 mg/L 6-BA + 2 mg/L 2,4-D) proved to be the most effective for callus induction (**Figure 5D**). To balance the screening efficiency and cytotoxicity, 50 mg/L hygromycin was determined to be the lowest lethal concentration for *P. ternata* callus (**Figure 5E**). The establishment of an efficient callus induction and screening system for *P. ternata* provides a foundation for subsequent genetic transformation.

### 3.9 Overexpression of *MAO*, *NCS I/II*, and *TyrAT* in *P. ternata* callus

**Figure 6:**
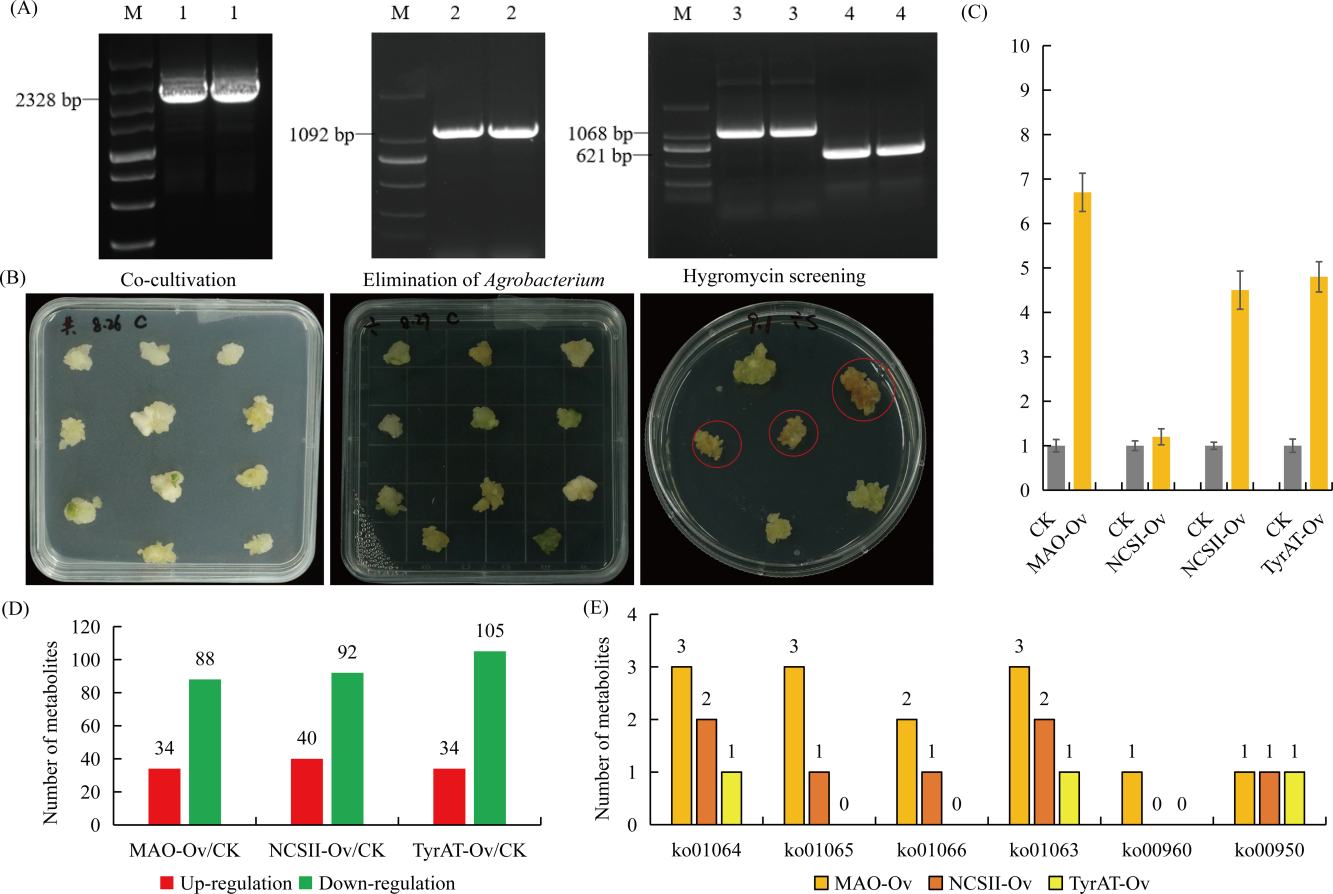
Effect of *MAO*, *NCS II*, and *TyrAT* genes on the alkaloid biosynthesis of *P. ternata.***(A)** Cloning of the full-length sequences of MAO, NCS I/II, and TyrAT genes. 1, MAO; 2, NCS I; 3, NCS II; 4, TyrAT. **(B)** The process of genetic transformation of *P. ternata* callus. **(C)** qRT-PCR analysis of the expression levels of the *MAO*, *NCS I, NCS II*, and *TyrAT* in *P. ternata* callus. A *P* value <0.05 was considered to be statistically significant and indicated by ‘*’. **(D)** The number of differentially accumulated metabolites in each comparison. **(E)** The number of alkaloid biosynthesis-related metabolites in each comparison.

A stable expression system was established to overexpress the *MAO*, *NCS I/II*, and *TyrAT* genes in *P. ternata* callus. Based on the transcriptome reference sequence, the full-length sequences of the *four* genes were identified and cloned via PCR amplification (**Figure 6A**). After 45 days of callus induction, fresh callus were harvested and subjected to Agrobacterium-mediated transformation. Co-cultivation of callus with over-expression vectors containing the target genes was performed to facilitate the infection process. After co-cultivation, the callus was transferred to a decontamination medium supplemented with 400 mg/L cefotaxime to eliminate Agrobacterium. Subsequently, positive callus were selected on medium containing 50 mg/L hygromycin (**Figure 6B**). qRT-PCR analysis demonstrated that the *MAO*, *NCS II*, and *TyrAT* genes were successfully overexpressed in *the transgenic callus lines* (**Figure 6C**).

The control (CK) and three independent overexpression (Ov) lines, including *MAO*-Ov, *NCSII*-Ov, and *TyrAT*-Ov, were harvested for untargeted metabolomic analysis. A total of 17,088 effective ion signals were detected by mass spectrometry. Quality control analysis demonstrated that the untargeted metabolomics experiment exhibited high repeatability and robust data quality. HMDB-based annotation identified 547 metabolites, including 207 lipids and lipid like molecules, 110 organic acids and their derivatives, and 58 organic heterocyclic compounds (**Supplementary Figure 3A**). KEGG enrichment analysis showed that most annotated metabolites were enriched in the shikimic acid alkaloid biosynthesis pathway (ko01063), guanine, lysine, and nicotinic acid alkaloid biosynthesis (ko01064), histidine and purine alkaloid biosynthesis (ko01065), terpenoid and alkaloid biosynthesis (ko01066), and the biosynthesis of various alkaloids (ko00996). These metabolomic data provide a foundation for elucidating the regulatory roles of *MAO*, *NCSII*, and *TyrAT* in alkaloid biosynthesis (**Supplementary Figure 3B**).

### 3.10 Effect of *MAO*, *NCS II*, and *TyrAT* genes on the alkaloid biosynthesis of *P. ternata*

A number of differentially accumulated metabolites were identified in each comparison, including 34 up- and 88 down-regulated metabolites in the *MAO*-Ov/CK comparison, 40 up- and 92 down-regulated metabolites in the *NCSII*-Ov/CK comparison, and 34 up- and 105 down-regulated metabolites in the *TyrAT*-Ov/CK comparison (**Figure 6D**). Then, we focused on six alkaloid biosynthesis-related KEGG pathways. Our data showed that the metabolic differences caused by *MAO*-Ov were the greatest, while those caused by TyrAT-Ov were the smallest. These results indicate that *MAO* is a potential key gene regulating alkaloid biosynthesis in *P. ternata* callus (**Figure 6E**).

Further analysis found that the three transgenic treatments (*MAO*-Ov, *NCSI*-Ov, and *TyrAT*-Ov) had significant effects on the accumulation of alkaloid precursors. In the *MAO*-Ov/CK comparison, succinic acid (1.45-fold, *P* < 0.05) and histamine (1.48-fold, *P* < 0.05) were significantly up-regulated; meanwhile, the levels of putrescine (0.65-fold, *P* < 0.005), malic acid (0.63 folds, *P* < 0.05), and 4-hydroxycinnamic acid (0.47-fold, *P* < 0.005) were significantly decreased. In the *TyrAT*-Ov/CK comparison, glutamic acid increased 3.62-fold, and the contents of 4-hydroxycinnamic acid and hydroxy-tryptamine were decreased significantly.

## 4 Discussion

As a significant traditional Chinese herb, PR possesses diverse pharmacological activities, such as cough suppression and antihypertensive effects. Alkaloids are the primary active ingredients of PR (Zhang et al., 2016). Numerous alkaloids were isolated from the tubers of *P. ternata* (Du et al., 2018; Chen et al., 2025). However, the complete alkaloid biosynthesis pathway in *P. ternata* has not been fully investigated.

In *P. ternata*, several transcriptomic analyses have been performed, identifying candidate genes involved in metabolite synthesis and organ formation (Huang et al., 2016; Zhang et al., 2016; Xue et al., 2019; Lu et al., 2020; Guo et al., 2022; He et al., 2022; Guo et al., 2023b; Yin et al., 2023). To reveal the significant regulatory genes responsible for tuber color variation, a total of 673,123 unigenes from 12 cDNA libraries were identified by Yin’s group (Yin et al., 2023). To identify the candidate genes involved in the biosynthesis of benzoic acid and ephedrine, 89,068 unigenes were identified by Zhang’s group (Zhang et al., 2016). To identify the key candidate genes involved in flavonoid biosynthesis, researchers identified a total of 121,812 unigenes from six libraries in *P. ternata* under heat stress (Guo et al., 2023a). In 2020, the transcriptomes of two varieties of *P. ternata* (T2 and T2Plus line) were characterized via *de novo* assembly, resulting in a total of 107,777 unigenes (Lu et al., 2020). In our study, we identified 63,106 *P. ternata* unigenes, indicating sufficient sequencing depth for functional gene discovery. In addition, our experiment represents the first systematic study into the effects of geographical origins on the secondary metabolism of *P. ternata* tubers. Based on a large number of unigenes, we conducted a preliminary exploration of the key candidate genes involved in alkaloid biosynthesis in *P. ternata* tubers.

The MIA biosynthetic pathway has been well-identified in the medicinal plant *Catharanthus roseus* (Li et al., 2023). Geranyl diphosphate (GPP), which serves as the entry product for the MIA pathway, is synthesized by GPPS. In our study, we identified six genes that encode GPPS in *P. ternata*, which is more than the number reported in *C. roseus* (Rai et al., 2013). Most of the GPPS encoding genes in *P. ternata* highly were expressed in the CX tubers, indicating a greater supply of precursors in these tissues. TDC is a pyridoxal 5’-phosphate-dependent enzyme that catalyzes the conversion of tryptophan to tryptamine, an important product of the MIA pathway (Qiao et al., 2022). In *P. ternata*, the majority of TDC encoding genes were found to be significantly expressed in the JZ tubers, suggesting a greater concentration of tryptamine in the JZ tubers. TyrAT is the initial enzyme involved in the metabolism of tyrosine, which serves as the entry point for the BIA pathway (Sun et al., 2023). Through transcriptomic analysis, a differentially expressed *TyrAT* gene (DN7072_c0_g1) was identified in the JZ tubers. COR catalyzes the final two steps in the biosynthesis of morphine, a specialized metabolite closely related to BIAs (Carr et al., 2021). In *P. ternata*, all six COR encoding genes were highly expressed in the CX tubers, indicating an active morphine biosynthesis pathway in the CX tubers. Our data showed that the *MAO*, *NCS I/II*, and *TyrAT* genes exhibited differential expression between *P. ternata* of JZ in Hubei and CX in Yunnan. Therefore, in our study, we focused on the *MAO*, *NCS I/II*, and *TyrAT* genes to conduct transgenic research.

Various TF families have been considered to be involved in different steps of secondary metabolite synthesis pathways (Feng et al., 2023; Yu et al., 2025). For example, the transcription factor EfABI4 is involved in controlling starch production in *Euryale ferox Salisb* seeds (Wu et al., 2022). VvibZIPC22 regulates the synthesis of flavonoids in grapes (Malacarne et al., 2016). CsAP2L1, CsWRKY1, and CsMYB1 are involved in the regulation of cannabinoid biosynthesis (Liu et al., 2021). Common TFs such as bHLH, WRKY, and MYB, which are involved in the regulation of alkaloid synthesis, have all been identified in *P. ternata* (Yamada et al., 2011; Yamada and Sato, 2013; Godbole et al., 2022). According to Nagi’s research, the TFs CgUPC2A and CgUPC2B control the genes responsible for ergosterol synthesis in *Candida glabrata* (Nagi et al., 2011). In our study, we identified a total of 445 TF genes belonging to 13 families, among which three ARF, two MYC, one MADS, two ZIP, three bHLH, four MYB, and three ERF family genes were identified as DEGs between the JZ and CX tubers. Our results provided various candidate regulatory factors involving in the alkaloid biosynthesis of *P. ternata* tuber.

BIAs are a diverse group of plant-derived compounds with essential pharmaceutical properties (Takenaka et al., 2024). In the BIA biosynthesis pathway, MAO is responsible for converting dopamine to 3,4-dihydroxyphenylacetaldehyde, a substrate for aldehyde dehydrogenase-2 (Yao et al., 2010). In *P. ternata*, over-expression of MAO affects the content of several alkaloid precursors, suggesting its important role in the regulation of alkaloid biosynthesis. NCS is the first enzyme that catalyzes the BIA skeleton formation (Xu et al., 2025). NCS I/II serves as the key rate-limiting enzyme in the BIA synthesis branch (Li et al., 2025). TyrAT catalyzes the transamination of L-Tyr and α-ketoglutarate, yielding 4-hydroxyphenylpyruvic acid and L-glutamate (Lee and Facchini, 2011). Through genetic transformation, we generated *MAO*, *NCS II*, and *TyrAT* overexpression callus lines, which laid a foundation for analyzing their functions. Our data indicated that *NCS* and *TyrAT* may play a role in the accumulation of alkaloid precursors, suggesting that there is a feedback regulation in alkaloid biosynthesis.

## 5 Conclusions

In our study, two *P. ternata* cultivars with distinct alkaloid profiles were used to analyze the genes involved in alkaloid biosynthesis pathways. Based on reference sequences, we predicted the rough alkaloid biosynthetic pathways and identified several key enzyme-encoding genes and transcription factor genes. Furthermore, stable overexpression of *MAO*, *NCS II*, and *TyrAT* in *P. ternata* callus significantly altered the accumulation of precursors for alkaloid biosynthesis. Our findings help to elucidate the alkaloid biosynthesis pathways in *P. ternata* tubers, which is essential for maintaining quality of PR.

## Data Availability

The data presented in this study are openly available in the NCBI Short Read Archive with accession number PRJNA989026 and the National Genomics Data Center with accession number OMIX012336.

## Supplementary material

Supplementary Table 1 The detailed information of the transcriptomic analysis.

Supplementary Table 2 The GO enrichment analysis of the DEGs.

Supplementary Table 3 The KEGG enrichment analysis of the DEGs.

Supplementary Table 4 Screening of differentially expressed TF genes.

Supplementary Figure 1 Multiple sequence alignment of the ITS region for *P. ternata* samples.

Supplementary Figure 2 Overview of the transcriptome datasets.

Supplementary Figure 3 HMDB- and KEGG-based annotation of all detected metabolites.
