# Supplementary material for: Identification and functional verification of key genes involved in alkaloid biosynthesis in Pinellia ternata
Source: Front Plant Sci. 2026 Apr 28;17:1737389. doi: 10.3389/fpls.2026.1737389 (PMC13161035; doi:10.3389/fpls.2026.1737389)
Supplement: Supplementary Figure 1 — Multiple sequence alignment of the ITS region for P. ternata samples. [file DataSheet1.pdf]

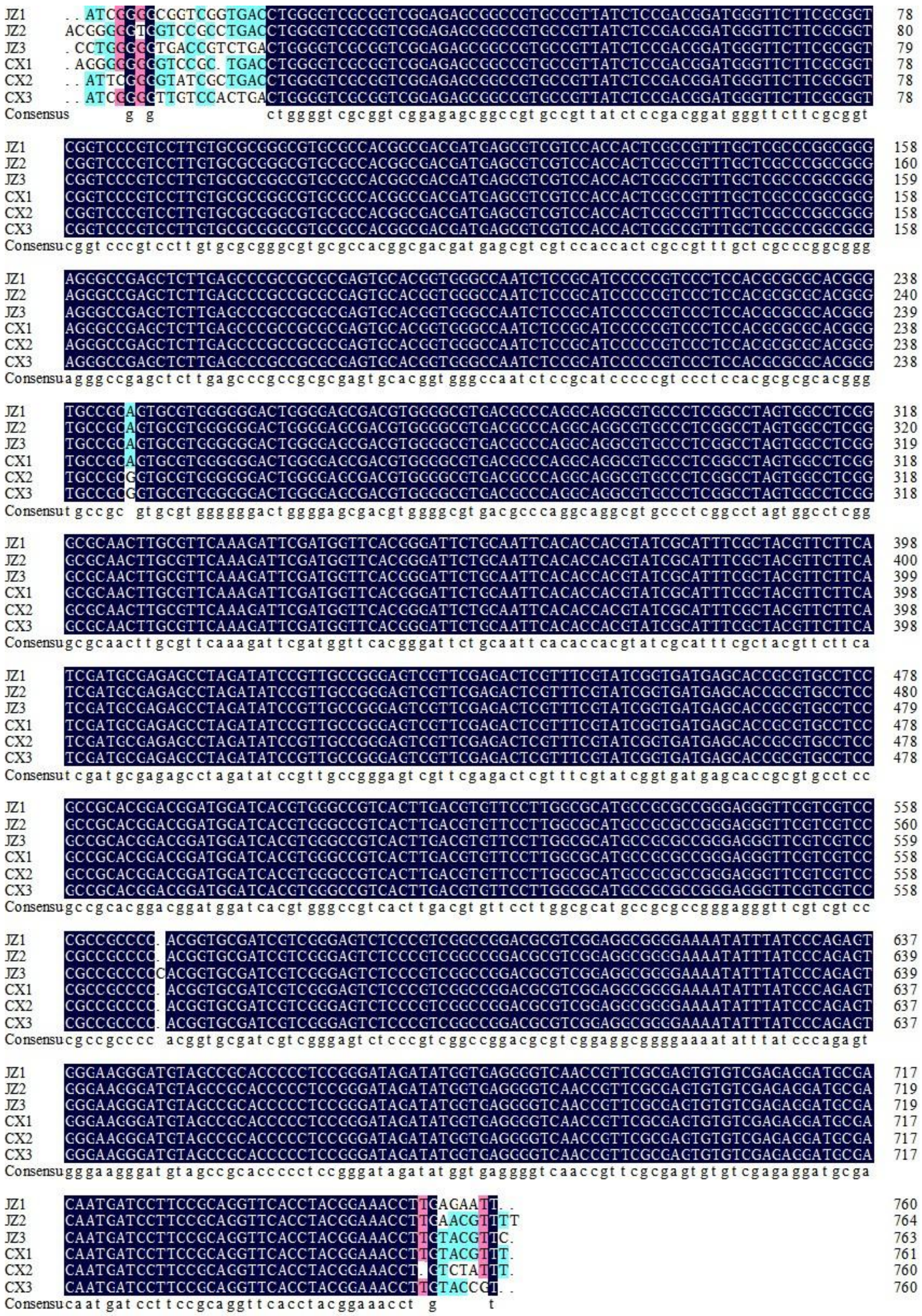

Figure S1 **Multiple sequence alignment of the ITS region for *P. ternata* samples.** The sequences of samples JZ1–3 and CX1–3 were aligned using DNAMAN. Dark blue shading indicates 100% sequence identity, while other colors represent nucleotide substitutions or variations. The consensus sequence is shown at the bottom. The alignment confirms that all samples share >98% homology, verifying their identity as *P. ternata*.
