# Supplementary material for: Identification and functional verification of key genes involved in alkaloid biosynthesis in Pinellia ternata
Source: Front Plant Sci. 2026 Apr 28;17:1737389. doi: 10.3389/fpls.2026.1737389 (PMC13161035; doi:10.3389/fpls.2026.1737389)
Supplement: Supplementary Figure 2 — Overview of the transcriptome datasets. [file DataSheet2.pdf]

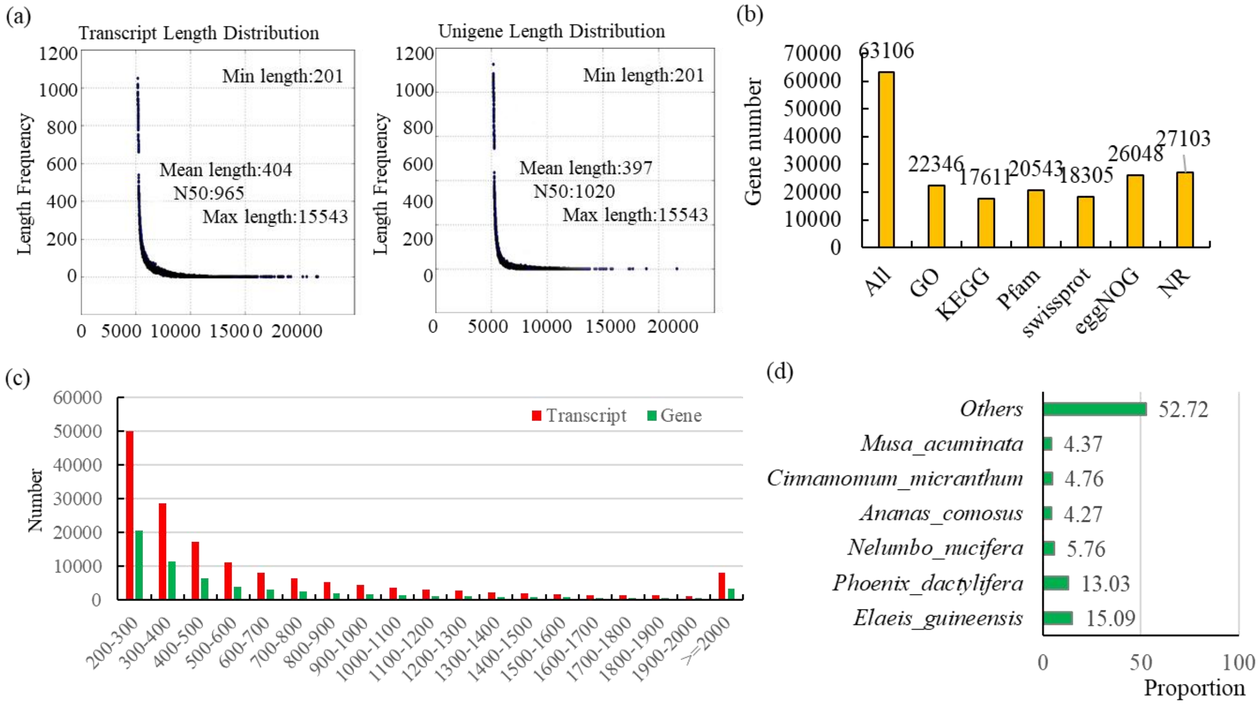

Figure S2 **Overview of the transcriptome datasets.** (a) Detailed information of all clean reads from each sample, which were combined and resulted in 159,135 transcripts and 63,106 unigenes. (b) The number of unigenes annotated by different databases, including Nr, Swissprot, KOG, KEGG, GO and Pfam. (c) The length distribution of assembled transcripts and unigenes in *P. ternata*. (d) Species distribution of all annotated unigenes.
