# Supplementary material for: Identification and functional verification of key genes involved in alkaloid biosynthesis in Pinellia ternata
Source: Front Plant Sci. 2026 Apr 28;17:1737389. doi: 10.3389/fpls.2026.1737389 (PMC13161035; doi:10.3389/fpls.2026.1737389)
Supplement: Supplementary Figure 3 — HMDB- and KEGG-based annotation of all detected metabolites. [file DataSheet3.pdf]

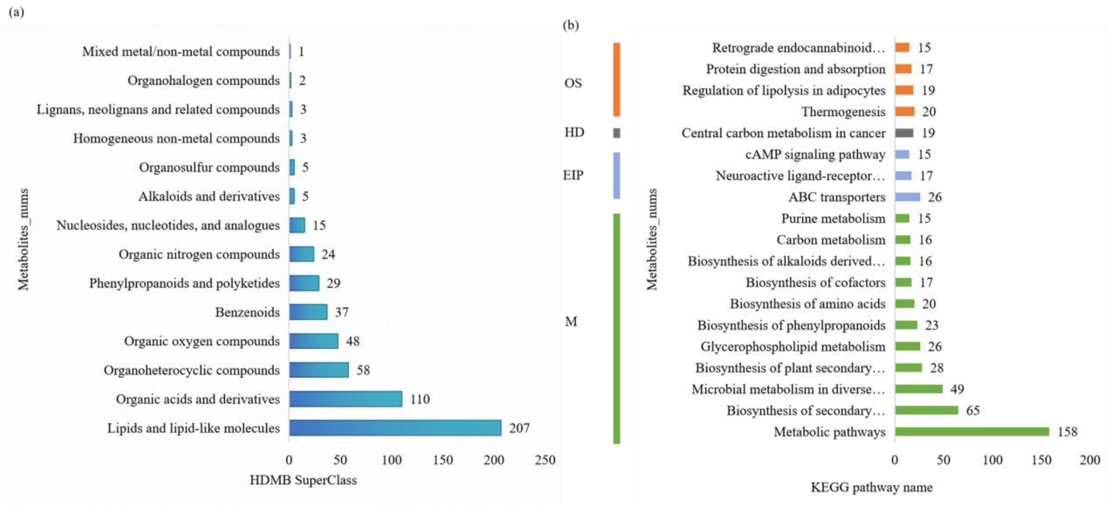

Figure S3 **HMDB- and KEGG-based annotation of all detected metabolites.** (a) Classification and Annotation of Metabolites in HMDB Database. (b) Classification and annotation of metabolites in the KEGG database. OS: Organismal Systems; HD: Human Diseases; EIP: Environmental Information Processing; M: Metabolism.
